# Argonaute 4 as an Effector Protein in RNA-Directed DNA Methylation in Human Cells

**DOI:** 10.3389/fgene.2019.00645

**Published:** 2019-07-04

**Authors:** Kanwalat Chalertpet, Piyapat Pin-on, Chatchawit Aporntewan, Maturada Patchsung, Praewphan Ingrungruanglert, Nipan Israsena, Apiwat Mutirangura

**Affiliations:** ^1^Interdisciplinary Program of Biomedical Sciences, Faculty of the Graduate School, Chulalongkorn University, Bangkok, Thailand; ^2^Faculty of Medicine, King Mongkut’s Institute of Technology Ladkrabang, Bangkok, Thailand; ^3^Department of Mathematics and Computer Science, Faculty of Science, Chulalongkorn University, Bangkok, Thailand; ^4^Center of Excellence in Molecular Genetics of Cancer and Human Diseases, Department of Anatomy, Faculty of Medicine, Chulalongkorn University, Bangkok, Thailand; ^5^Stem Cells and Cell Therapy Research Unit, Faculty of Medicine, Chulalongkorn University, Bangkok, Thailand; ^6^Department of Anatomy, Faculty of Medicine, Chulalongkorn University, Bangkok, Thailand

**Keywords:** human Argonaute 4, human RNA-directed DNA methylation, human RdDM, CPP-AGO4, epigenomic editing

## Abstract

DNA methylation of specific genome locations contributes to the distinct functions of multicellular organisms. DNA methylation can be governed by RNA-dependent DNA methylation (RdDM). RdDM is carried out by endogenous small-RNA-guided epigenomic editing complexes that add a methyl group to a precise DNA location. In plants, the Argonaute 4 (AGO4) protein is one of the main catalytic components involved in RdDM. Although small interfering RNA or short hairpin RNA has been shown to be able to guide DNA methylation in human cells, AGO protein-regulated RdDM in humans has not yet been evaluated. This study aimed to identify a key regulatory AGO protein involved in human RdDM by bioinformatics and to explore its function in RdDM by a combination of AGO4 knockdown, Alu small interfering RNA transfection, AGO4-expressing plasmid transfection, chromatin immunoprecipitation, cell-penetrating peptide-tagged AGO4 combined Alu single-guide RNA transfection, and methylation analyses. We found that first, human AGO4 showed stronger genome-wide association with DNA methylation than AGO1–AGO3. Second, endogenous AGO4 depletion demethylated DNA of known AGO4 bound loci. Finally, exogenous AGO4 *de novo* methylated the bound DNA sequences. Therefore, we discovered that AGO4 plays a role in human RdDM.

## Introduction

DNA methylation plays critical regulatory roles in both of prokaryotic (Adhikari and Curtis, 2016) and eukaryotic (Tang et al., 2012; Zhang et al., 2018) organisms, including mammals (Jones and Takai, 2001). In humans, specific methylation controls cellular processes involved in many characteristics of multicellular organisms, such as tissue-specific expression and differentiation (Khavari et al., 2010; Wan et al., 2015), genomic imprinting, X-chromosome inactivation (Chen and Li, 2006; Fedoriw et al., 2012), and silencing of transposable element transcription (Hollister and Gaut, 2009; Rerkasem et al., 2015; Anwar et al., 2017). For the past decade, a connection has been reported between small RNAs and specific DNA methylation modifications, which results in transposable element control and genomic stability through RNA-mediated transcriptional gene silencing (Morris, 2008; Moazed, 2009).

RNA-mediated transcriptional gene silencing, a fundamental mechanism in the regulation of gene expression, has been extensively studied over the past several years. One mechanism of RNA-mediated transcriptional gene silencing is RNA-directed DNA methylation (RdDM), which was first discovered in plants and described as noncoding RNA-mediated epigenetic modification (Wassenegger et al., 1994). Studies in plants have found that one of the key proteins playing a role in RdDM is Argonaute 4 (AGO4) (He et al., 2009; Lahmy et al., 2016). RdDM has been further explored in humans, and it has been revealed that many varieties of small noncoding RNAs, such as small interfering RNA (siRNA) and short hairpin RNA (shRNA), can contribute to RdDM (Morris et al., 2004; Castanotto et al., 2005; Patchsung et al., 2018); however, the process has not been well characterized. Here, we investigated whether human AGO4 has a plant-like RdDM function and hypothesized that the human AGO4 protein might be involved in an increase in DNA methylation levels that participates in RdDM.

Mechanistically, RdDM is mainly involved in the biogenesis of 24-nucleotide siRNAs and the loading of single-guide RNAs (sgRNAs) onto the AGO4 protein. sgRNA-bound AGO4 then recruits DRM2 to promote *de novo* methylation at sites of sgRNA homology (Zilberman et al., 2003; Xie and Yu, 2015). Hence, to investigate RdDM pathways in humans, we initially focused on identifying the AGO protein that affects RdDM and validating the results in AGO-binding genes. The human AGO subfamily is composed of AGO1 to AGO4, each of which exhibits different functions, abundances, and levels of expression in cells (Sasaki et al., 2003; Schürmann et al., 2013). Notably, the structures of these proteins are similar and are mostly conserved; their structures are composed of N-terminal domains (involved in RNA cleavage), PAZ domains (which bind to the 3’-ends of small RNAs), MID domains (which function in small RNA loading and bind to the 5’-ends of small RNAs), and PIWI domains (containing RNase H-like active sites for RNA cleavage) (Swarts et al., 2014).

However, some distinctions of human AGO have been noted. AGO4 differs in a few amino acid sequences from the N-terminal and PIWI domains of AGO2, which has been well described with respect to its structure and function. AGO4 does not exhibit catalytic cleavage activity (Hauptmann et al., 2014), and the quantity of AGO4 and its expression at both the mRNA and protein levels are the lowest among its protein family (Valdmanis et al., 2011; Turchinovich et al., 2016).

Interspersed repetitive sequences consisting of the long and short interspersed elements (LINEs and SINEs) were chosen for primary observation according to a previous study showing that siRNA produced from inverted repeats and the AGO4 protein can cause maintenance of DNA methylation in plants (Zilberman et al., 2004). Moreover, studies in humans have found that LINE-1, from which bidirectional transcripts are produced to form siRNA, can suppress LINE-1 retrotransposition through DNA methylation (Yang and Kazazian, 2006; Chen et al., 2012). Nevertheless, Alu siRNA transfection can also induce Alu methylation (Patchsung et al., 2018).

In the current study, we performed a genome-wide association study and revealed that human AGO4 colocalizes to sites of promoter methylation. Furthermore, we investigated DNA methylation changes under conditions in which the AGO4 protein was depleted or upregulated. Our findings would help to extend the understanding of epigenetic pathways in humans.

## Materials and Methods

### Colocalization Between Promoter Methylation and Argonaute Proteins

We performed a whole-genome colocalization analysis between a promoter methylation dataset (GSE20598) (Komashko and Farnham, 2010) and AGO-binding sites (CLIPZ database) (Khorshid et al., 2010). The GSE20598 dataset provided the amount of promoter methylation data in human embryonic kidney (HEK293) cells. The CLIPZ database provided AGO-binding locations in the same cell line. Based on these two sources of data, the correlations between promoter methylation and AGO proteins were identified.

### GSE20598 Dataset

The GSE20598 dataset was obtained through employed chromatin immunoprecipitation (ChIP)-chip promoter microarray (GPL6603) analysis based on the HG17 genome build. Only one sample (GSM517330), consisting of 5-meC MeDIP DNA from HEK293 cells treated with 50% acetic acid (control), was used in our analysis. A total of 15 methylation probes were assigned to each gene and were tiled over approximately 1.5 kb across a promoter. The probes were 50 bp in length. The amount of methylation at a promoter was determined from the summary of all 15 probes.

### CLIPZ Database

The CLIPZ database lists all the known binding sites of AGO proteins in the entire genome of HEK293 human embryonic kidney cells. The database contains two important files: mapped sequences of RNA sequences bound by Argonaute proteins (AGO1–4) and genomic maps of the locations of these RNA sequences in the whole genome.

Mapping began at chromosome 1 and was stopped for RNA sequences that could be mapped to >30 locations (mostly repeat sequences). The AGO protein family members are AGO1, AGO2, AGO3, and AGO4. We downloaded the following files from http://test.mirz.unibas.ch/smirnaWeb/geneBio/smiRNA/temp/10544043421949953483/samples in the following subfolders (October, 2011):

AGO1:    /230/mapped_sequences,/230/genome_mappings
AGO2:    /238/mapped_sequences,/238/genome_mappings
AGO3:    /239/mapped_sequences,/239/genome_mappings
AGO4:    /240/mapped_sequences,/240/genome_mappings

### LiftOver Tool

The ChIP-chip promoter microarray used in GSE20598 was based on the HG17 genome build, whereas the CLIPZ database was based on the HG18 genome build. Therefore, the genomic locations from HG17 were converted to locations from HG18 using LiftOver software (http://genome.ucsc.edu/cgi-bin/hgLiftOver).

### NimbleScan Software

In the GSE20598 dataset, the methylation level at each promoter was summarized to a single value. In fact, the microarray used a total of 15 probes tiled over a promoter. To assess the methylation level of each probe, the supplementary file (GSE20598_RAW.tar) from Gene Expression Omnibus was needed (Barrett et al., 2009). NimbleScan software (version 2.6) from the manufacturer of the microarray (Roche NimbleGen) was used to process the supplementary file (http://www.nimblegen.com/downloads/support/NimbleScan_v2p6_UsersGuide.pdf).

### Promoter Selection

Only promoters that were bound with only one type of AGO protein (AGO1 or AGO2 or AGO3 or AGO4) were considered in our analysis to avoid any possible interactions between AGO proteins. The boundary of a promoter was defined as the region between the first and the 15th (the last) probes of each promoter.

### Distance Between Argonaute Binding Sites and Methylation Sites

Both AGO-binding sites and methylation sites (or probes) were considered as numerical ranges in base pair. The distance between these sites was defined as the distance between the centers of two ranges. Each probe could contain multiple CpG sites, but the distribution of CpG sites on all probes was symmetric. Thus, we assumed that the methylation sites were located at the center of the probes.

### Association of Argonaute-Binding Sites and Methylation Levels

The association between AGO-binding sites and methylation levels was identified by 2 × 2 contingency tables. A contingency table counted the number of methylation probes. The first and the second column counted the number of probes with high (MET+) and low (MET-) methylation levels. The first and the second rows counted the number of probes that were in proximity with AGO-binding sites (AGO+) and the probes that were in AGO-depleted regions (AGO-). Each contingency table yielded odds ratio (OR), 95% confidence interval (CI), and a Chi-squared *p*-value.

### Establishment of AGO4sh and Tetracycline Resistance Knocked-Down Cell Lines

To generate a lentivirus vector encoding AGO4sh, a double-stranded AGO4sh (target sequence 5’-GGCCAGAACTAATAGCAATT-3’) was cloned into pENTR^™^/H1/TO (Invitrogen, CA, USA) and subcloned into the pLenti4/BLOCK-iT^™^-DEST Gateway^®^ Vector (Invitrogen) using LR Clonase^®^ II (Invitrogen). Lentiviruses expressing AGO4sh and tetracycline resistance (TetR) were generated according to the manufacturer’s instructions. Briefly, 293FT cells were transfected with 5 µg of pLenti4/H1/TO/shAGO4 (for AGO4sh), pLenti6/TR (for TetR), and 5 µg of each packaging plasmid (pLP1, pLP2, and pVSV-G) (Invitrogen) using the FugeneHD transfection reagent (Roche, Basal, Switzerland). After transfection for 48 h, the media were collected and filtered through a 0.45-µm-pore-size filter. The virus-containing supernatants were centrifuged at 25,000 rpm for 90 min. Viral pellets were resuspended with Opti-MEM (Invitrogen). HEK293 cells were transduced with lentivirus-expressed shAGO4 and TetR and supplemented with 6 µg/ml polybrene (Sigma-Aldrich, MO, USA). After transduction for 48 h, the cells were dissociated and seeded in 10-cm^2^ dishes at 10% confluence. The cells were then cultured under Zeocin^™^ (100 µg/ml) (Thermo Scientific, MA, USA) selection for 3 weeks. Zeocin^™^ and blasticidin-resistant clonal isolates were tested for AGO4sh activity in a tetracycline-regulated manner using real-time PCR. HuSH shRNA plasmid, pGFP-C-shLenti plasmid (Origene, MD, USA) was used as a plasmid control according to the manufacturer’s instructions.

### Cell Culture and Treatment

For the AGO4sh and TetR knockdown cell lines, AGO4sh-regulated HEK293 cells were maintained in Dulbecco’s modified Eagle’s medium (DMEM) (Thermo Scientific) supplemented with 2 mM L-glutamine (Sigma-Aldrich), 10% (v/v) heat-inactivated fetal bovine serum (FBS) (Thermo Scientific), 10 mg/ml antibiotic/antimycotic (Thermo Scientific), 500 µg/ml zeocin at 37°C, and 5% (v/v) CO_2_. To knock down AGO4 protein expression, AGO4sh-HEK293 cells were treated with 2 µg/ml tetracycline for 9 days (Tet on or Tet+). AGO4sh-HEK293 cells (not treated with tetracycline or Tet off or Tet-) were also harvested on seventh and ninth day. Media and tetracycline were changed every 3 days later. All cultures were treated with 5’-azacytidine (5-AC) (Sigma-Aldrich) on days 7 to 9, which was changed every day. Cell cultures of the HeLa cervical adenocarcinoma cell line were purchased from the American Type Culture Collection (ATCC, VA, USA). These cells were cultured in DMEM containing 10% FBS (Thermo Fisher Scientific) and 1% antibiotic–antimycotic (Thermo Scientific) in a 5% CO_2_ incubator.

### Protein Preparation and Western Blot Analysis

AGO4- and PC-overexpressing HeLa cells were harvested as whole cell lysates using radio immunoprecipitation assay lysis buffer (Amresco, OH, USA). The cells were further sonicated at a 30% amplitude for 5 s, which was repeated three times. Then, the protein concentration was determined by using a BCA Protein Assay Kit (Thermo Scientific) according to the manufacturer’s instructions. Forty micrograms of whole cell lysate was subsequently used for sodium dodecyl sulfate (SDS) polyacrylamide gel electrophoresis and Western blot analysis as previously described (Fuks et al., 2000). The antibodies employed in these assays were 1:1,000 eLF2C4 (C-12) (SC-32665) (Santa Cruz, CA, USA), 1:5,000 goat anti-mouse IgG-HRP (SC-2005) (Santa Cruz), and an anti-beta actin antibody [AC-15] (HRP) (Ab49900) (Abcam, Cambridge, UK). Each antibody was diluted in the BlØk^®^ Noise Cancelling Reagent (Merck Millipore, MA, USA). Finally, the signals were visualized using the SuperSignal^®^ west femto chemiluminescent substrate (Thermo Scientific) on a C-DiGit^®^ blot scanner (LI-COR Biosciences, NE, USA).

### Chromatin Immunoprecipitation

To detect AGO4 protein-binding genes, ChIP was performed as previously described with some modifications (Boyd and Farnham, 1999). Cells were fixed with 1% final concentration of formaldehyde for 30 min at room temperature, and then glycine was added at a final concentration of 0.125 M for 5 min at room temperature to stop the reaction. Next, the cells were washed twice with 1X protease inhibitor (Thermo Scientific) containing cold phosphate-buffered saline and collected using cell scrapers. Thereafter, the cells were sonicated at a 30% amplitude five times; the supernatant was collected by centrifugation and diluted using ChIP dilution buffer. Protein A or G plus-agarose (Santa Cruz, CA, USA) was added to the samples for 1 h at 4°C on a rotating shaker for preclearing, and the supernatant was harvested to perform immunoprecipitation using 10 µg of each of the HA-probe (Y-11) (SC-805) (Santa Cruz) and normal rabbit IgG (#2729) (Cell Signaling, MA, USA) antibodies. Protein A or G plus agarose was added again to precipitate the antigen–antibody complexes, and the precipitated complexes were washed with 150 and 500 mM sodium chloride (NaCl) and lithium chloride buffer, respectively. The complexes were then decrosslinked, and the DNA was precipitated using ethanol and purified by phenol–chloroform DNA extraction. Finally, the DNA was used for further experiments consisting of real-time PCR or conventional PCR.

### DNA Preparation and Sodium Bisulfite Treatment for Methylation Detection

Cells were harvested by trypsinization, and their DNA was extracted using 10% sodium dodecyl sulfate (SDS) (Sigma-Aldrich), lysis buffer II (0.75 M NaCl, 0.024 M ethylenediaminetetraacetic acid at pH 8), and 20 mg/ml proteinase K (USB, OH, USA); the cells were incubated at 50°C for three nights for cell digestion. Phenol/chloroform extraction and ethanol precipitation were then carried out as previously described (Sambrook, 1989). Next, 750 ng of each DNA sample was subjected to sodium bisulfite treatment using the EZ DNA methylation-Gold^™^ kit (Zymo Research, CA, USA) according to the manufacturer’s instructions. The eluted DNA was subsequently used for pyrosequencing or combined bisulfite restriction analysis (COBRA).

### Pyrosequencing to Determine DNA Methylation Levels

Sodium bisulfite-treated DNA was used to perform PCR to enrich the DNA template for pyrosequencing (Colella et al., 2003). Pyrosequencing was carried out using the PyroMask^®^ Gold Q24 machine (Qiagen, Hilden, Germany) according to the manufacturer’s protocol. Pyrosequencing primers were subsequently designed focusing on 3-5 targets CpG dinucleotides of *TRDP*, *C16ORF89*, *ATAT1*, and *MSN*. All primer sequences are depicted in **Supplementary Table 1**. Each sample was analyzed in triplicate for this experiment. In brief, 20-µl aliquots of the PCR products were immobilized on streptavidin Sepharose HP beads (GE Healthcare, Little Chalfont, UK) and purified using a PyroMark Q24 vacuum workstation (Qiagen) according to the manufacturer’s instructions. Then, the samples were denatured at 80°C for 2 min and annealed to a sequencing primer. Thereafter, the samples were subjected to analysis in a pyrosequencing machine. Nucleotide dispensation order and sequence analyses were performed with PyroMark Q24 software 2.0.6 (Qiagen). The pyrograms were analyzed in CpG assay mode to quantify the methylation percentage of each CpG.

### Alu siRNA Transfection

As previously described (Patchsung et al., 2018), Alu siRNA (sense, 5’-CUUUGGGAGGCCGAGGCGGGCGGAUCA-3’; antisense, 5’-AUCCGCCCGCCUCGGCCUCCCAAAGUG-3’) was used to transfect the Tet+/Tet–treated AGO4sh knockdown cell lines. Cells were cultured with Tet (Tet+ or AGO4-) or without Tet (Tet- or AGO4+) for 7–9 days. One day before transfection, the treated and untreated cells were seeded into a 24-well plate at 5 × 10^4^ cells. Alu siRNA was then transfected to cells using Lipofectamine^®^ 2000 (Invitrogen) for 48 h. The cells were collected to observe methylation changes by COBRA.

### LINE-1 or Alu-Combined Bisulfite Restriction Analysis

To observe the methylation levels of LINE-1 or Alu in transfected cells, sodium bisulfite-treated DNA from each sample was amplified by using 1× PCR buffer (Qiagen), 0.2 mM deoxynucleotide triphosphate (Biotechrabbit, Hennigsdorf, Germany), 1 mM magnesium chloride (Qiagen), 25 U of HotStarTaq DNA Polymerase (Qiagen), and 0.3 µM of the LINE-1 met FW, RW or Alu met FW, RW primers, as shown in **Supplementary Table 2**. For LINE-1 amplification, the cycling program was set at 95°C for 15 min, followed by 30 cycles of 95°C for 45 s, 55°C for 45 s, 72°C for 45 s, with a final extension 72°C for 7 min. The LINE-1 PCR products were then subjected to COBRA by using 2 U of TaqI (Thermo Scientific) with 0.5× TaqI buffer (Thermo Scientific) and incubated at 65°C overnight. For Alu amplification, the program was set as follows: 95°C for 15 min for one cycle and 40 cycles of 95°C for 45 s, 57°C for 45 s, 72°C for 45 s, with a final extension 72°C for 7 min. The Alu PCR products were subjected to COBRA using 2 U of TaqI (Thermo Scientific), 2 U of TasI (Thermo Scientific) with 5× nuclear extraction buffer 3 (New England Biolabs, MA, USA), and 1 µg/µl bovine serum albumin (New England Biolabs) and were incubated at 65°C overnight. Then, the cut PCR products were analyzed in 8% acrylamide gels with SYBR gel stain (Lonza, Basel, Switzerland). The band intensity of LINE-1 or Alu methylation was observed and measured by Strom840 and ImageQuanNT software (Amersham Biosciences, Little Chalfont, UK) (Kitkumthorn and Mutirangura, 2011).

### Methylation Analysis

For LINE-1 methylation analysis, the band intensities of LINE-1 products with sizes of 92, 60, 50, 42, and 32 bp were applied for the calculation of LINE-1 methylation levels according to the following formula: [(A + 2C + F) × 100]/(2A + 2B + 2C + 2F), where A = band intensity at 92 bp/92, B = 60/56, C = 50/48, D = 42/40, E = 32/28, and F = [(D + E) − (B + C)]/2. For Alu methylation analysis, the band intensities of five Alu products with sizes of 133, 90, 75, 58, and 43 bp were used for the calculation of Alu methylation levels according to the following formula: [(2F + D + C) × 100]/(2A + 2C + 2D + 2F), where A = band intensity at 133/133, B = 58/58, C = 75/75, D = 90/90, E = 43/43, F = [(E + B) − (C + D)]/2. The global methylation of LINE-1 or Alu measured in each replication was then used for statistical analysis using a paired-sample *T*-test.

### Argonaute 4 Plasmid Transfection

Overexpression of AGO4 was performed by the using pIRESneo-FLAG/HA AGO4 plasmid (Addgene, MA, USA). One day before transfection, HeLa cells were seeded at 2 × 10^5^ cells/ml in 6-well plates in 2-ml DMEM containing 10% FBS without antibiotics. The following day, plasmid transfection complexes were prepared; 2 µg of the AGO4 plasmid and the pcDNA3.1/myc-HIS A empty plasmid (PC) (Invitrogen) were transfected into HeLa cells with 10 µl of Lipofectamine^®^ 2000 (Invitrogen) mixed with Opti-MEM^®^ reduced serum media (Invitrogen) in a total volume of 500 µl according to the manufacturer’s protocol. The transfection duration was 72 h for the observation and quantification of LINE-1 or Alu methylation; overexpression of AGO4 mRNA was also confirmed.

### RNA Preparation and cDNA Synthesis

Cells were harvested using trypsinization and washed with phosphate-buffered saline, after which Trizol^™^ (Invitrogen) was added to the cell pellet according to the manufacturer’s protocol. Then, 500–1,000 ng of total RNA from each sample was used to synthesize cDNA using the RevertAid^™^ first-strand cDNA synthesis kit (Thermo Scientific) according to the manufacturer’s specification.

### Real-Time PCR

To observe AGO4 mRNA expression and to quantify, AGO4-bound LINE-1 or Alu DNA levels, real-time PCR was carried out using Power SYBR^®^ Green PCR master mix (Applied Biosystems, CA, USA) with AGO4 FW, RW (GAPDH FW, RW was used as an internal control); copy LINE-1 FW, RW; or copy Alu FW, RW (shown in **Supplementary Table 2**). The amplifications were performed within a 7,500-fast real-time PCR system (Applied Biosystems). The ΔΔCT method (Livak and Schmittgen, 2001) was used to calculate the amount of AGO4-bound LINE-1 or Alu in AGO4 plasmid overexpressing cells compared with pcDNA3.1 empty plasmid-transfected cells. Paired sample T-tests were performed for statistical analysis at CIs of 95%.

### Cell-Penetrating Peptide Argonaute 4 Plasmid Construction and Protein Production

To produce cell-penetrating peptide AGO4 (CPP-AGO4) and CPP- enhanced green fluorescent protein recombinant proteins, 9× arginines (R9) as CPP and AGO4 mRNA (NM_017629.3) were in-frame inserted into the pRSET A vector (Thermo Scientific) between *Bam*HI and *Eco*RI sites. Both vectors were constructed by the GeneArt^™^ Plasmid Construction Service (Thermo Scientific). The fidelities of the sequences were confirmed by Sanger sequencing.

The CPP-AGO4 vector was transformed into BL21(DE3)pLysS competent cells (Promega, WI, USA) for CPP-AGO4 protein production. The cells were then cultured in 2YT medium, and overexpression was induced by 1 mM isopropyl-1-thio-β-D-galactopyranoside (Sigma-Aldrich) until the OD600 reached 0.8. The cells were cultured further for 20 h at 20°C in a 250-rpm incubation shaker. Then, the cells were collected by centrifugation at 10,000 rpm for 15 min at 10°C. Cell pastes were lysed by lysis buffer [50 mM Tris-HCl (pH 8.0), 300 mM NaCl, 10 mM imidazole, 10% glycerol, 10 mg/ml lysozyme, 1% Triton X-100, and 1× protease inhibitor] and sonicated and centrifuged at 10,000 rpm at 10°C for 20 min to harvest the crude protein supernatant. CPP-AGO4, which was soluble in supernatant, was purified on a HisTrap^™^ HP (GE Healthcare) column according to the manufacturer’s instructions. Purified CPP-AGO4 protein was confirmed using Coomassie^®^-Brilliant-Blue-R-250 (C.I.42660) (Merck Millipore)-stained 6% SDS polyacrylamide gel electrophoresis. Furthermore, Western blot analysis was performed using 1:2500 AGO4 (D10F10) (Cell Signaling), 1:5,000 anti-rabbit IgG, HRP-linked [#7074] (Cell Signaling) or 1:2,500 anti-6X HIS tag^®^ [HIS.H8] (ab18184) (Abcam), and 1:5,000 anti-mouse IgG, HRP-linked [#7076] (Cell Signaling) antibody. Concentrations of both purified proteins were determined by Pierce BCA protein assay kit (Thermo Scientific).

### Cell-Penetrating Peptide Argonaute 4-Alu Transfection

The purified CPP-AGO4 was conjugated to sgRNA of Alu (5’-AUCCGCCCGCCUCGG CCUCCCAAAG-3’) using a slicing assay as previously described (De and MacRae, 2011) with some modifications. Briefly, 10 µg of CPP-AGO4 protein was mixed with 200 nM of sgRNA in conjugation buffer [1.5 mM MgCl_2_, 5% glycerol, 50 mM KCl, and 20 mM Tris-HCl (pH 7.0)] and incubated in a 37°C water bath for 30 min. Then, the conjugated complexes of CPP-AGO4-sgRNA were dropped into 500 µl DMEM-cultured 7.5 × 10^4^ cells in a 24-well plate to allow the complexes to be internalized into the cells and induce methylation at specific targets homologous to the sgRNA; non-induced CPP-AGO4 represented as buffer was used as a control. After transfection for 72 h, cells were collected to measure changes of methylation levels of target genes.

## Results

### Argonaute 4 is Related to Promoter Methylation

**Figure 1:**
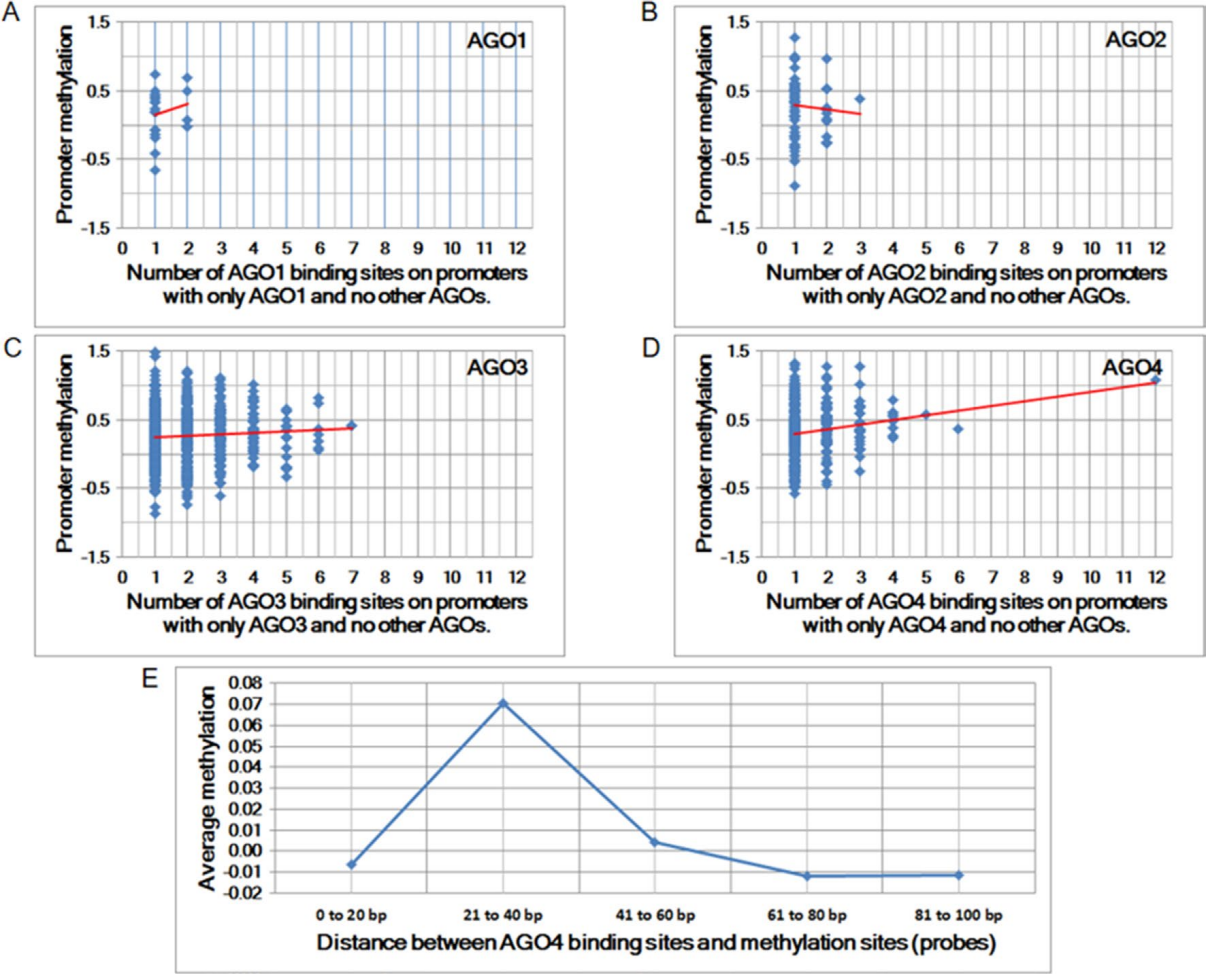
The correlation between Argonaute (AGO) proteins and promoter methylation. **(A** to **D)** Promoter methylation vs. the number of AGO-binding sites. The Pearson correlation coefficients for A to D are 0.01, −0.06, 0.06, and 0.17, and the corresponding *p*-values are 8.63E-01, 6.51E-01, 1.11E-01, and 1.48E-03, respectively. **(E)** Average methylation vs. proximity to AGO-binding sites. Each column represents the average amount of methylation at a single probe if the proximity is within a predefined distance.

To determine whether any AGO protein is associated with DNA methylation, we first performed whole-genome comparisons between the promoter methylation dataset (GSE20598) and AGO-binding sites (CLIPZ database) (Komashko and Farnham, 2010; Khorshid et al., 2010). The GSE20598 dataset provided the amount of DNA methylation in HEK293 cells; the CLIPZ database provided AGO-binding locations in the same cells. On the basis of these two datasets, the association between DNA methylation and AGO proteins was identified. Among all AGO proteins, AGO4 showed the strongest correlation with DNA methylation, as summarized from multiple probes (Pearson correlation coefficient = 0.17 and *p*-value = 1.48 × 10^−3^) (**Figures 1A–D**).

The increase in the number of AGO4-binding sites in a promoter is proportional to the amount of methylation at the promoter. Moreover, the binding position of AGO4 is correlated with the methylation of a single probe (**Figure 1E**). Sequence logos and positions are reported in relation to the transcriptional start sites (TSS) of AGO4 binding and methylation (**Supplementary Figure S1**). Interestingly, AGO4 binds to the sense strand of methylated DNA located immediately downstream of the TSS (**Supplementary Figure S2**).

**Figure 2:**
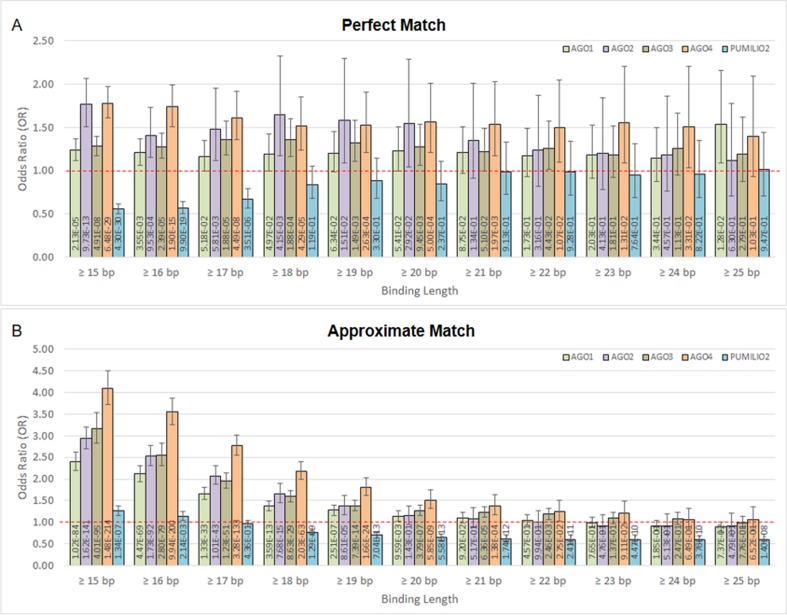
The association between methylation levels and protein-binding sites. The Chi-square *p*-values are calculated from 2 × 2 contingency tables that count the number of methylation probes. The rows of a contingency table separate highly and lowly methylated probes, whereas the columns separate probes that are in proximity with protein-binding sites and probes that are not. Note that a single probe might be in proximity with multiple protein. **(A)** Only ChIP DNAs that are perfectly matched with the human genome are included. **(B)** Only ChIP DNAs that are approximately matched with the human genome are included. This also includes perfectly matched DNAs.

Moreover, we found that DNA methylation is associated with AGO-binding sites (**Figures 2A, B**). The highly methylated probes are in proximity with the binding sites of AGO1, AGO2, AGO3, and AGO4 proteins. This is indicated by odds ratio (OR) and 95% confidence interval (CI) > 1. In contrast, the number of association studies of the control protein, PUMILIO2, shows OR and CI <1. In **Figure 2A**, it demonstrated OR and CI when the ChIP sequences that are perfectly matched with the human genome were counted, whereas **Figure 2B** reported approximate match. Other similar sequences contain mismatch, insertion, and deletion being alternative targets. All four AGOs (AGO1, AGO2, AGO3, and AGO4) showed OR and CI >1 in several tests. However, AGO4 showed the higher OR and the lowest *p*-value in most tests (**Figure 2** and **Supplementary Table 3**). By allowing approximate match, AGO4 shows the highest OR at binding length ≥15 bp, OR = 4.09, 95% CI = 3.73 to 4.50, *p*-value = 1.48E-214 (**Figure 2B** and **Supplementary Table 3**).

### The Argonaute 4 Protein is Involved in *De Novo* Methylation of Argonaute 4-Binding Genes

**Figure 3:**
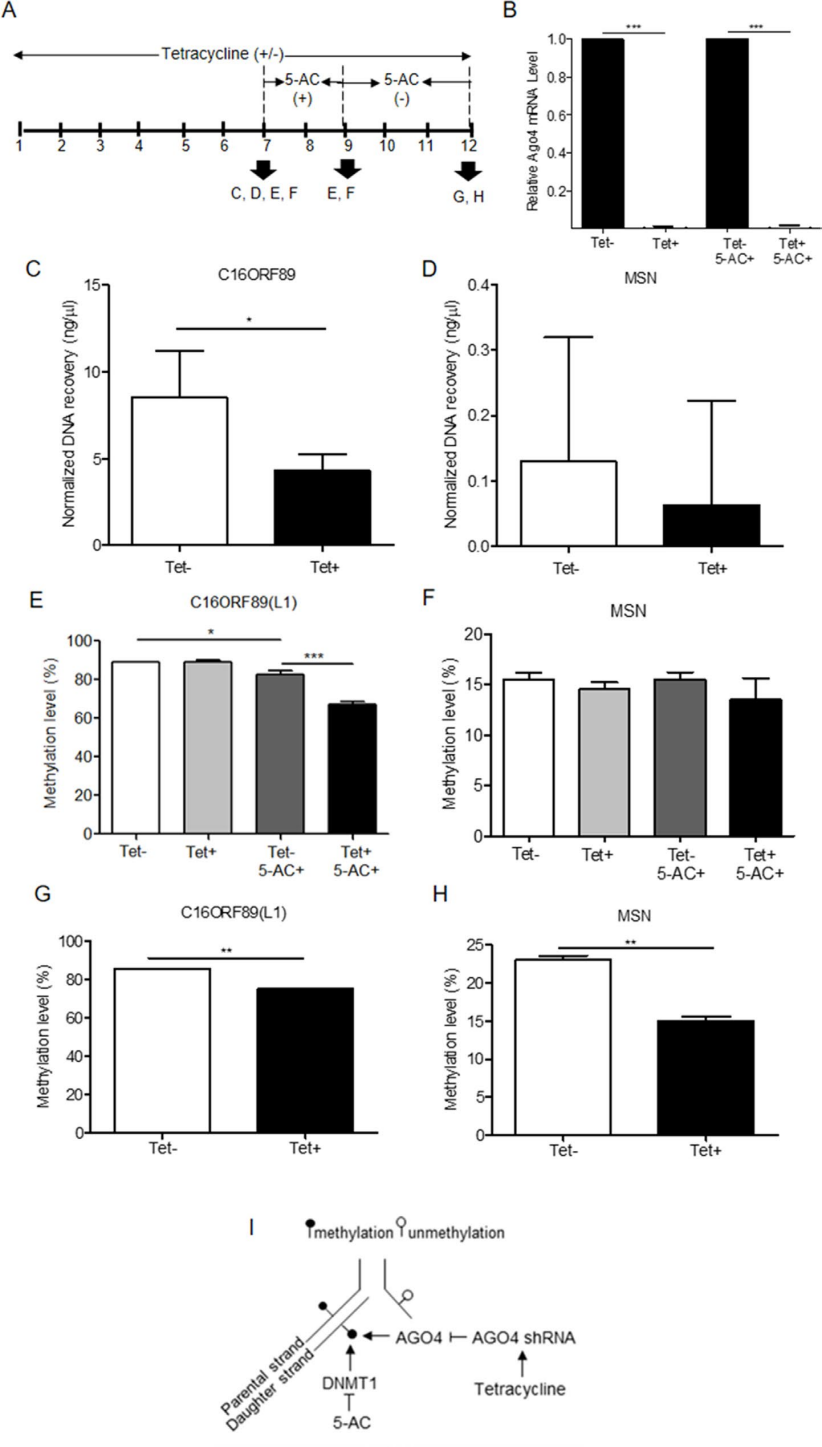
Association of the AGO4-binding genes and methylation levels. **(A)** Scheme showing that tetracycline-treated cells switch off AGO4 protein expression (Tet+) from day 1 to 12 and that 5’-azacytidine treatment from day 7 to 9 inhibits DNA methyltransferase (5-AC). **(B)** Detection of AGO4 mRNA in Tet- (AGO4 expression) and Tet+ (No AGO4 expression) cells; AGO4 was significantly expressed in the absence of Tet. **(C)** Confirmation of AGO4-binding genes showed that AGO4 only bounds to the selected gene from bioinformatics data as exemplified by *C16ORF89*. **(D)** However, AGO4 did not bind to a gene lacking the AGO4-binding site, *MSN*. **(E)** The AGO4 methylation level of the gene, which contains AGO4-binding sites, was increased due to the presence of the AGO4 protein (Tet-). This increased methylation level was not associated with the presence of DNA methyltransferase (5-AC+). **(F)** These results were not observed for a gene lacking AGO4-binding sites, *MSN*. **(G** to **H)** Recovery of DNA methylation levels is found in cells expressing the AGO4 protein (Tet-) with 5’-azacytidine withdrawal in both of AGO4-binding gene, *C16ORF89* and non-AGO4-binding gene, *MSN*; AGO4 is inferred to methylate previously demethylated loci. The above data are the representative dataset from three independent experiments presented as the mean ± SEM. Statistical analyses were performed using a paired-sample *t*-test, where *p* < 0.05, *p* < 0.01, and *p* < 0.005 are represented as *, **, and ***, respectively, where Tet+ means tetracycline treatment, 5-AC+ means azacytidine treatment, and Tet- and 5-AC- mean untreated groups (I). Schematic overview of the role of AGO4 in *de novo* methylation. After DNA replication, DNA methylation is maintained by DNMT1 by the recognition of hemimethylated CpG on the parental strand as a template and the addition of newly methylated CpG to the daughter strand. DNMT1 is inhibited by the action of 5-AC, and the methylation level should be decreased by half under 5-AC treatment. In our study, when tetracycline was added, AGO4 shRNA was manipulated, resulting in AGO4 expression repression. Thus, without tetracycline, AGO4 is upregulated and binds to DNA loci, and DNA methylation is maintained, although 5-AC is added, suggesting that AGO4 involved in *de novo* methylation

To investigate AGO4-binding genes and the role of AGO4 in DNA methylation, we obtained target genes from analyses of the GSE20598 and CLIPZ databases. These bioinformatics data provided us with a large number of genes that show a connection between AGO4-binding and promoter methylation. We randomly selected eight loci of three genes: *TRDP, C16ORF89*, and *ATAT1* that have high level of DNA methylation and AGO4 binding to study the interaction between AGO4 and DNA methylation. *MSN*, which lacks AGO4 binding, was used as a negative control. The experiments were carried out by depleting endogenous AGO4 using a tetracycline-regulated shRNA-expression system in the HEK293 cell line (AGO4sh). The timing of tetracycline (Tet) and 5-AC treatment is shown (**Figure 3A**). Depletion of AGO4 mRNA expression was confirmed (**Figure 3B**). After the cells were cultured with Tet (Tet+) or without Tet (Tet-) for 7 days, they were collected to confirm the AGO4-binding genes. All four selected genes bound large quantities of AGO4; *C16ORF89* is provided as an example (**Figure 3C**); the other genes showed similar results (**Supplementary Figures S3A, B**), with the exception of *MSN* (**Figure 3D**). Methylation levels were also evaluated; however, there was no significant difference in methylation levels between the Tet+ and Tet- cells at all nine loci of the four known AGO4-binding genes or *MSN* (**Figures 3E, F** and **Supplementary Figures S4A–G** at Tet- and Tet+ bars). Thereafter, the HEK293 cell line was treated with a combination of Tet and 5-AC to limit AGO4 mRNA and inactivate DNA methyltransferase activity for two additional days, until day 9; methylation changes were then investigated. From days 7 to 9, the methylation levels of all loci were significantly reduced in Tet+ and 5-AC+ shAGO4-induced cells compared with Tet- and 5-AC+ cells (**Figures 3E, F** and **Supplementary Figures S4A–G** at Tet- 5-AC+ and Tet+ 5-AC+ bars). This finding suggested a role of AGO4 in the demethylation of AGO4-bound sequences.

The mechanism by which AGO4 prevented demethylation by 5-AC suggested a role of AGO4 in *de novo* DNA methylation. To observe the *de novo* methylation function of endogenous AGO4, cells were cultured continuously with Tet and 5-AC for 9 days. Then, the cells were cultured for 3 days (days 9 to 12) without 5-AC and with or without Tet. The methylation levels of all loci were recovered in cells lacking tetracycline treatment (Tet-, 5-AC+) (**Figures 3G, H** ****and **Supplementary Figures S5A–G**). Moreover, tetracycline and scramble shRNA by themselves did not influence DNA methylation levels significantly (**Supplementary Figure S6**). This finding means that AGO4 reintroduces DNA methylation to previously demethylated loci, as shown in **Figure 3I**.

### Argonaute 4 as a Master Protein Regulates Human RNA-Dependent DNA Methylation

**Figure 4:**
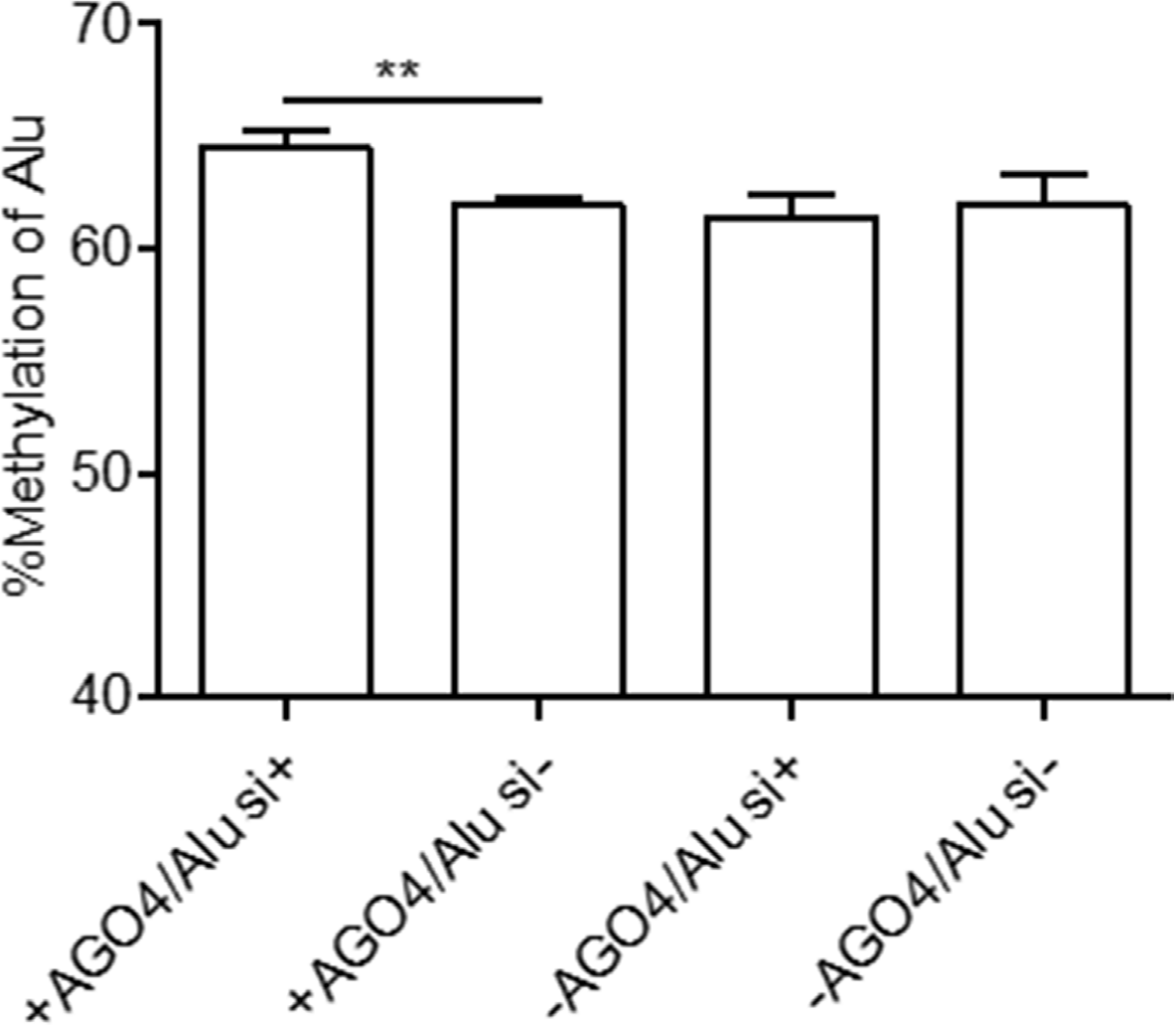
Alu methylation upon Alu siRNA transfection in Tet-controlled AGO4-expressing cells. The Alu methylation level was significantly increased when AGO4 was upregulated under Alu siRNA transfection. The above data are the representative dataset from six independent experiments presented as the mean ± SEM. Statistical analysis was performed using an unpaired *t*-test where *p* < 0.01 is represented as **.

Previously, we demonstrated that Alu element siRNA (Alu siRNA) transfection increased Alu methylation (Patchsung et al., 2018). To determine whether siRNA can promote methylation in human cells and whether this process is dependent on AGO4, Alu siRNA was used to transfect the AGO4sh-induced HEK293 cell line, and the cells were cultured under Tet+ or Tet- conditions. We found that Alu siRNA increased the Alu methylation level when AGO4 was not limited; on the other hand, Alu siRNA did not promote methylation when AGO4 was depleted in Tet+-cultured HEK293 cells (**Figure 4**). Therefore, Alu siRNA may form an RdDM complex with AGO4 to methylate ALU sequences.

**Figure 5:**
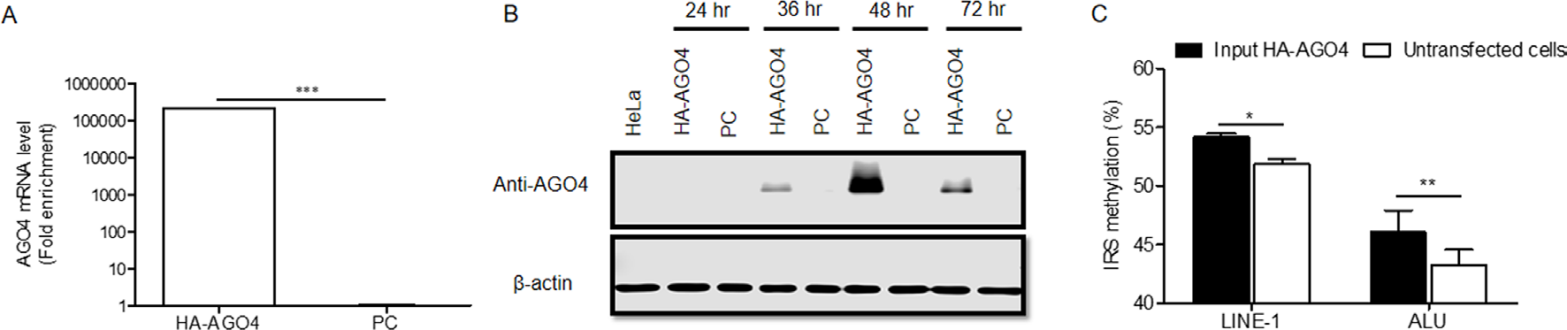
Overexpression of HA-AGO4 increases global genomic methylation. **(A)** Confirmation of AGO4 mRNA expression in AGO4- or PC-overexpressing cells after transfection for 72 h by using real-time PCR showed that AGO4 mRNA was significantly upregulated in AGO4-overexpressing cells, and **(B)** Western blot analysis showed the expression of the HA-AGO4 protein in a time-course experiment. β-Actin was used to confirm equal protein loading of each lane. AGO4 protein expression was at the highest level at 48 h after transfection. **(C)** Overexpression of HA-AGO4 resulted in a significant increase in interspersed repetitive sequence methylation in the input (cell lysate) HA-AGO4 compared with untaransfected HeLa cells, detected at 72 h post-transfection. The above data in A and C are the representative dataset from five independent experiments presented as the mean ± SEM. Statistical analyses were performed using a paired *t*-test where *p* < 0.05, *p* < 0.01, and *p* < 0.005 are represented as *, **, and ***, respectively.

**Figure 6:**
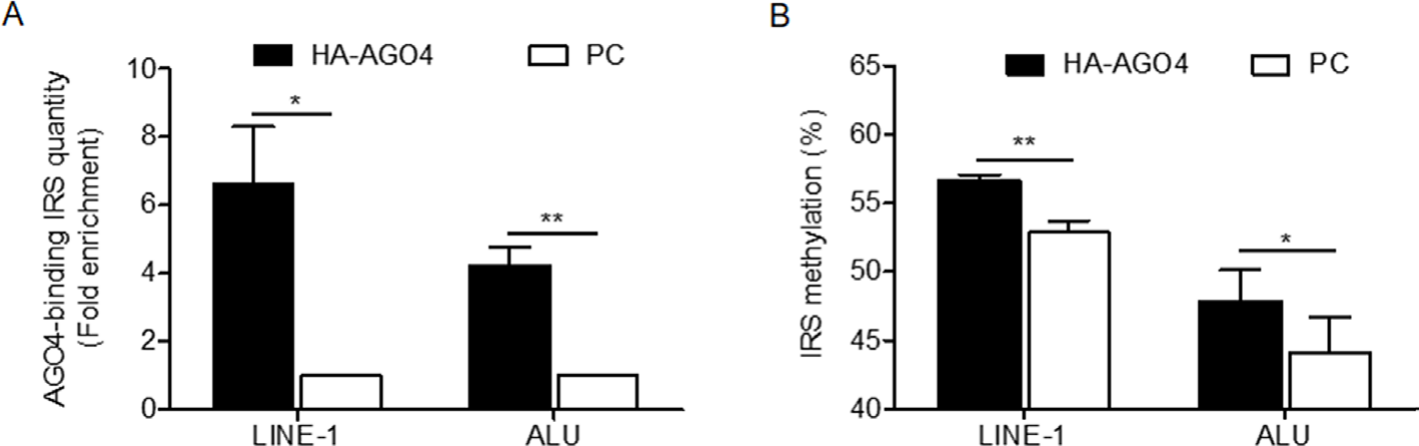
AGO4 localization is involved in RdDM by introducing genomic DNA methylation in human cells. **(A)** ChIP experiments using a hemagglutinin (HA) antibody showed elevation of the levels of AGO4-binding LINE-1 or ALU found in AGO4-overexpressing cells compared with their control counterparts. **(B)** Moreover, the binding of the AGO4 protein to interspersed repetitive sequence increased DNA methylation.

To determine whether human AGO4 is a main protein in the RdDM complex and directly methylates DNA, we transfected a hemagglutinin (HA)-tagged AGO4 plasmid into HeLa cells and evaluated whether the HA-AGO4 protein could methylate DNA. We also used an empty plasmid (pcDNA, or PC more briefly) as a control plasmid. Overexpression of AGO4 after transfection was detected after 72 h at the mRNA level and at 24, 36, 48, and 72 h at the protein level (**Figures 5A, B** and **Supplementary Figure S7**). At 72 h post-transfection, the genome-wide interspersed repetitive sequence methylation of both LINE-1 and Alu was observed (**Figure 5C**). Then, AGO4 localization was observed using an HA antibody in a ChIP experiment. We discovered that AGO4-HA preferentially bound to LINE-1 and Alu sequences (**Figure 6A**), which were highly methylated (**Figure 6B**).

**Figure 7:**
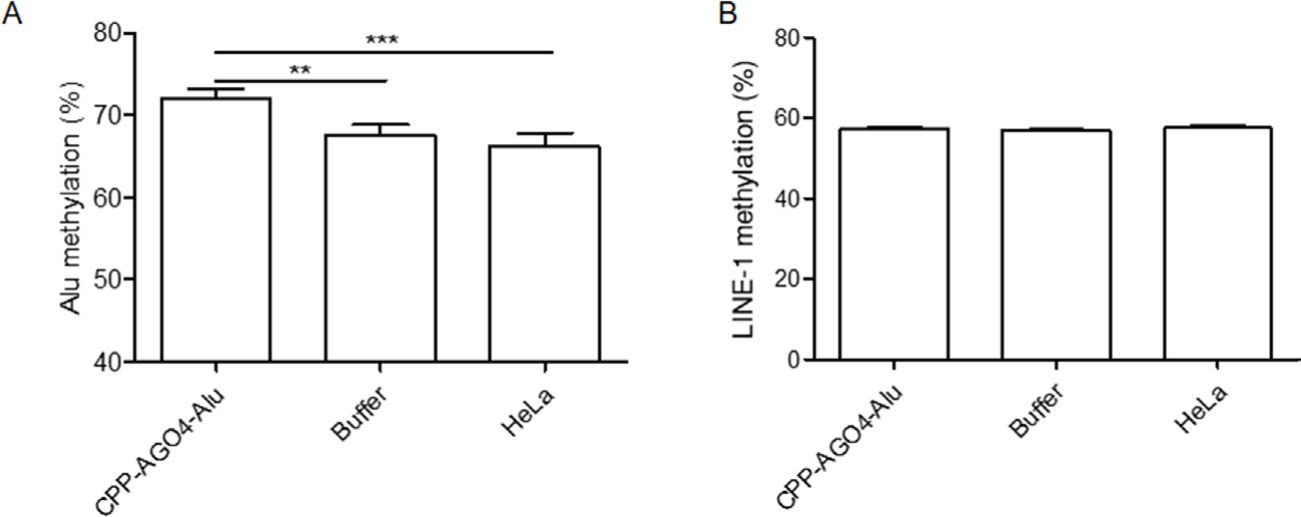
AGO4 protein specifically induced DNA methylation of target loci. By using CPP-AGO4 conjugated with Alu sgRNA (CPP-AGO4-Alu) transfected to HeLa cells, we found that **(A)** Alu methylation level was significantly increased in CPP-AGO4-Alu transfected cells, while Alu methylation was not promoted in control counterpart groups. **(B)** While LINE-1 methylation level was not changed, this means the complex has no off targets to LINE-1. The representative dataset was obtained from five independent experiments. Statistical analyses were performed using a paired-sample *T*-test where *p* < 0.05 and *p* < 0.01 are represented as * and **, respectively, where buffer means non-inducible CPP-AGO4 transfection.

To date, the technology of using cell-penetrating peptides (CPP) is developed to deliver biological cargoes to target cells (Madani et al., 2011) and to apply as therapeutic molecules for clinical application (Copolovici et al., 2014). By this advancement, we constructed arginine-rich CPP-tagged AGO4 and conjugated it with Alu sgRNA. The complex was transfected to cells, aimed to promote Alu methylation. We found that the result was consistent when CPP-AGO4-Alu was transfected to HeLa cells; Alu methylation was augmented significantly in CPP-AGO4-Alu-transfected cells but not in their control counterparts (**Figure 7A**). LINE-1 methylation level was studied to evaluate off targets, and we found that LINE-1 methylation level was not increased by CPP-AGO4-Alu-transfected cells (**Figure 7B**). Therefore, HA-AGO4 *de novo* methylates LINE-1 and Alu sequences, as well as CPP-AGO4-Alu can promote Alu methylation with no off-target effects.

## Discussion

In this study, we performed several experiments to determine that human AGO4 is a crucial protein in the RdDM complex. First, we found AGO protein locational overlap with DNA methylation. Second, the combination of AGO4 downregulation and 5-AC treatment can demethylate AGO4-bound loci. Third, AGO4 expression is required for Alu methylation upon Alu siRNA transfection. Finally, AGO4 overexpression and AGO4 protein transfection increase DNA methylation of AGO4-bound sequences.

Referring to our bioinformatics study, we used Pearson correlation coefficients and the 2 × 2 contingency tables to evaluate the correlation of promoter methylation and the number of AGO-binding sites. We found that both of these statistical tools did not provide much significant difference between each human AGO. Even a low-positive correlation and a large deviation were found. Nevertheless, AGO4 showed the most significant *p-values* and the highest OR. Therefore, we hypothesized that human AGO4 plays a role in RdDM. Our study showed that AGO1–AGO3 are also statistically significant, however, with lesser degree than AGO4 (**Figure 2A, B**). The RdDM function of human AGO4 might be redundant with other AGOs. This may explain why AGO4 double knockout mice are viable except for detection in male fertility (Modzelewski et al., 2012).

Neither AGO4 downregulation nor 5-AC treatment alone could effectively demethylate AGO4-bound loci. These findings suggested that AGO4 downregulation and 5-AC treatment demethylated DNA by different mechanisms. 5-AC functions as a DNA-hypomethylating agent by inhibiting human DNA methyltransferase 1 (DNMT1). DNMT1 plays a role in the maintenance of DNA methylation by recognition of hemi-methylated CpG and replication of CpG methylation to the opposite DNA strand (Stresemann and Lyko, 2008). Thus, 5-AC treatment should cause the level of DNA methylation to decrease after each round of DNA replication. In the present study, it was found that 5-AC could not reduce the methylation level when AGO4 was not downregulated. Therefore, AGO4 may play a role in *de novo* methylation and is not exclusively involved in DNA methylation maintenance, being inhibited by 5-AC (**Figure 3I**). Moreover, here, we demonstrated that newly synthesized AGO4 proteins methylated new loci. First, upregulated exogenous AGO4 increased Alu and LINE-1 methylation. Second, the AGO4 proteins, synthesized after stopping AGO4 shRNA induction, increased *MSN* methylation. We postulated that recently translated AGO4 proteins may bind to free small RNA that normally did not load onto AGO4. As demonstrated by **Figure 2B**, the binding of AGO4 did not require the perfect match; for that reason, this increased the possibility for newly forming AGO4 loading small RNA complex to methylate new loci.

In addition to confirming whether AGO4 is involved in RdDM, Alu siRNA transfection was performed. The increase in Alu methylation was significant only when AGO4 was present, verifying that AGO4 forms an RdDM complex together with siRNA. Moreover, overexpression of exogenous AGO4 also resulted in apparently higher methylation levels of LINE-1 and Alu, including CPP-AGO4-Alu transfection that can specifically promote Alu methylation; this finding is understandable because LINE-1 and Alu contribute a large number of endogenous siRNAs (Hadjiargyrou and Delihas, 2013).

Human RdDM complexes should be further explored in detail. In plants, there are two effector AGO proteins that are involved in RdDM, one of which is AGO4 (Matranga et al., 2005). AGO4/siRNA complexes are selectively transported into the nucleus and then recruit *de novo* methylase DRM2 to methylate target DNA (Ketting, 2011; Hegge et al., 2018). As noted before, DRM2 plays a role in plant RdDM and is an ortholog of human DNMT3, which is in accordance with the results we obtained showing that AGO4 participates in *de novo* methylation. Therefore, the association of RdDM effector protein complexes, including AGO4, each DNMT (DNMT3A/3B, and DNMT1), and other proteins that serve as transcription factors should be investigated. Another issue that might be a piece of the puzzle is that plant RdDM is processed by RNA polymerase IV and V (Pol IV and Pol V). These enzymes have conserved amino acid sequences similar to Pol II, but their largest domain is different from Pol II, suggesting that this domain might have evolved to function in RdDM (Huang et al., 2009). However, Pol I, II, and III are present in humans (Vannini and Cramer, 2012), and the specialized function of Pol involved in RdDM should therefore be identified. Moreover, evidence from *Schizosaccharomyces pombe* and human cells showed the presence of AGO1-mediated heterochromatin formation during transcriptional gene silencing, together with small RNAs (Kim et al., 2006; Qi et al., 2006); similarly, AGO3 is loaded by PIWI-interacting RNAs derived from transposon mRNAs in *Drosophila* leading to the hypothesis that AGO3-generating PIWI-interacting RNAs could play a role in chromatin formation at transposon loci (Ye et al., 2012). Thus, it is possible that human AGO1 and AGO3 could have interrelated effects on the methylation levels of histones and DNA. All of these observations give rise to one last question that should be addressed: is there any interplay between DNA methylation and chromatin modification caused by cohesion of AGO proteins? Notably, our genome-wide correlation study showed that the correlation between AGO2 and methylation was far less significant than that of the other AGO protein; thus, AGO2 may not play a role in RdDM and may only function in RNAi processes only. Furthermore, a study in mice indicated by generating individual AGO-deficient cells, each AGO possesses miRNA silencing activity. However, AGO2 yielded the greatest effect (Su et al., 2009). Consistent with the results of PR-9 anti-gene transfection in AGO-depleted cells, AGO2-lacking cells showed the largest decrease in PR-9 gene expression, while other AGOs had little effect (Chu et al., 2010). As the greatest quantity of AGO2 was found in humans, all of these lines of evidence led us to address whether the amount of AGO protein in cells could be a factor determining the pathway being used for gene silencing.

DNA methylation plays roles in several processes, such as genomic imprinting, X-inactivation, cell differentiation, aging, and adaptation to the environment. Our genome-wide study of colocalization between AGO4 binding and DNA methylation revealed that human AGO4 methylates DNA located immediately downstream of the TSS (**Supplementary Figure 2**). This location was also identified in our previous tissue-specific DNA methylation study (Muangsub et al., 2014), suggesting that the tissue specificity might be modulated by RdDM. However, as AGO4 can also bind both LINEs and SINEs and contribute to the control of gene expression and prevention of genomic instability (Mutirangura, 2019), other functions of AGO4 should also be explored.

Moreover, AGO has been proposed as a genomic editing tool in prokaryotes (Hegge et al., 2018). To date ZFNs, TALENs, CRISPR-cas9, and DNMT3A with a catalytically inactive Cas9 have been developed for future personalized medicine at both the genomic and epigenomic levels (Gaj et al., 2013; Liu et al., 2016; Vojta et al., 2016). Epigenomic editing could be beneficial in *ex vivo* or *in vitro* therapy. Therefore, CPP-AGO4, which is a human protein, might pave the way to be used as a new platform of therapies using protein transduction technology in the future. By this technology, it can avoid some drawbacks of using transgene technology, for instance, an insertion of recombinant DNA into host genomes that might lead to unwanted mutations (Bachu et al., 2015).

In summary, human AGO4 plays a role in siRNA-mediated DNA methylation, implying that RdDM exists in humans and is involved in *de novo* methylation. Further studies are needed to observe the roles of RdDM in human cells, and this study might shed light on the use of human AGO4 for future applications in epigenomic therapy.

## Data Availability Statement

All datasets generated for this study are included in the manuscript and the supplementary files.

## Author Contributions

KC performed all experiments related to AGO4 transfection and and wrote the manuscript. PP performed all Tet-on related experiments and wrote this part of the manuscript. CA performed all of the bioinformatics analyses and wrote this part of the manuscript. MP performed Alu siRNA experiment. PI and NI prepared Tet-on cells. AM conceived and designed the study, analyzed the data, and wrote and edited the manuscript.

## Funding

This work was supported by the Thailand Research Fund [DPG5980005, RSA5980060]; a 2019 Research Chair Grant from the National Science and Technology Development Agency, Thailand; the Anantara Siam Bangkok Hotel, Four Seasons Hotel Care for Cancer Fun Run in coordination with the Thai Red Cross Society; the Chulalongkorn University 100^th^ Year Birthday Anniversary Doctoral Degree Scholarship; the Chulalongkorn Academic Advancement in Its 2^nd^ Century Project; and the Thailand Research Fund through the Royal Golden Jubilee Ph.D. Program [PHD/0131/2554 to MP (student) and Professor AM (advisor)].

## Conflict of Interest Statement

The authors declare that the research was conducted in the absence of any financial relationships that could be construed as a potential conflict of interest.

## Supplementary Material

Table S1 Oligonucleotide sequences used in pyrosequencing.

Table S2 Oligonucleotide sequences and conditions for PCR and qPCR analyses.

Table S3 Contingency tables, odds ratios (ORs), 95% confidence intervals, and *p*-values.

Figure S1 Distribution of DNA bases on methylation and AGO4-binding sites.

Figure S2 Distribution of AGO4-binding sites on gene promoters.

Figure S3 Proving of AGO4-binding genes.

Figure S4 Confirmation of AGO4 protein associated to methylation level in AGO4-binding genes, independently function to DNA methyltransferase (DNMT).

Figure S5 Recovery of methylation level in some gene-containing AGO4-binding sites, in concordance with the presence of the AGO4 proteins after withdrawal of 5’-azacytidine.

Figure S6 Tetracycline (Tet) (the first panel), and 5’-azacytidine and combined tetracycline and 5’-azacytidine treatment (the second panel) in scramble shRNA-transfected HEK293 cells.

Figure S7 AGO4 protein expression in AGO4 plasmid and scramble shRNA-transfected and -untransfected HEK293 cells at 48 h after transfection.
