# Supplementary material for: Argonaute 4 as an Effector Protein in RNA-Directed DNA Methylation in Human Cells
Source: Front Genet. 2019 Jul 4;10:645. doi: 10.3389/fgene.2019.00645 (PMC6620710; doi:10.3389/fgene.2019.00645)
Supplement: Table S1 — Oligonucleotide sequences used in pyrosequencing. [file DataSheet_1.pdf]

SUPPLEMENTARY FILE

1. Supplementary tables
2. Supplementary figures legends

1. Suppplementary tables

Table S1 Oligonucleotide sequences used in pyrosequencing.

**PCR primers**		
**Primer**	**Sequences (5’-3’)**	**Annealing temperature (°C)**
TRDP-FW<br>TRDP-RW-biotin	AGAAGGTATTGTTGGTGTTATAGG<br>CCTCATTTTTTTTACTCTCCCTTACT	55
C16ORF89-FW<br>C16ORF89-RW-biotin	GGGTGGTTTGGGTAAAGGA<br>ACAACCCTCTCCCCAACA	56
ATAT1-FW<br>ATAT1-RW-biotin	GGGTGTGTAGGGATTGTGTAT<br>AAACATACCACAAAAAAAAACTCTCTA	56
MSN-FW<br>MSN-RW-biotin	TTGTTTAGAGAAGGAAAAGATAGGTAGTGA<br>CTCCTCTCCCACCCTAATCACAA	59
**Sequencing primers**	**Sequences (5’-3’)**	
TRDP	GGTTGGTTTTTGGATTATAA	-
C16ORF89	AAGGGTGGTTGTAGG	-
ATAT1	GTGAAGTTTTTGGAGTAAT	-
MSN	GAGTGGGGGTGGGGT	-

Table S2 Oligonucleotide sequences and conditions for PCR and qPCR analyses.

Primer	Sequences (5'-3')	Annealing temperature (°C)
LINE-1 met FW<br>LINE-1 met RW	GTTAAAGAAAGGGGTGAYGGT<br>AATACRCCRTTTCTTAAACCRATCTA	55
Alu met FW<br>Alu met RW	GGYGYGGTGGTTTAYGTTTGTAA<br>CTAACTTTTTATATTTTTAATAAAAACRAAATTTCACCA	57
AGO4-FW<br>AGO4-RW	CAGGAATTCAGGGAACCAGCCG<br>CTGCCTTCCGCACTGTCATGATC	56
GAPDH-FW<br>GAPDH-RW	TGGAAGGACTCATGACCACAG<br>TTCAGCTCAGGGATGACCTT	56°C – 60
Copy LINE-1 FW<br>Copy LINE-1 RW	CTCCCAGCGTGAGCG<br>ACTCCCTAGTGAGATGAACCCG	56
Copy Alu FW<br>Copy Alu RW	GGCTCACGCCTGTAATCCCAGC<br>GACGGGGTTTCACCATGTTGGCC	69

Table S3 Contingency tables**,** odds ratios (ORs)**,** 95% confidence intervals, and *p*-values. Each 2x2 table shows the association between methylation levels and protein binding sites. **(A)** Only ChIP DNAs that are perfectly matched with the human genome are included. **(B)** Only ChIP DNAs that are approximately matched with the human genome are included. This also includes perfectly matched DNAs.

Perfect match

Binding Length	Protein	a	b	c	d	OR	95 %CI	*p*-value	Binding Length	Protein	a	b	c	d	OR	95 %CI	*p*-value
15	AGO1	5,562	560	15,866	1,978	1.24	1.12 - 1.37	2.13E-05	21	AGO1	923	91	20,505	2,447	1.21	0.97 - 1.51	8.75E-02
	AGO2	2,496	176	18,932	2,362	1.77	1.51 - 2.07	9.73E-13		AGO2	307	27	21,121	2,511	1.35	0.91 - 2.01	1.34E-01
	AGO3	7,603	762	13,825	1,776	1.28	1.17 - 1.40	4.91E-08		AGO3	1,141	112	20,287	2,426	1.22	1.00 - 1.49	5.10E-02
	AGO4	6,476	497	14,952	2,041	1.78	1.61 - 1.97	6.48E-29		AGO4	719	56	20,709	2,482	1.54	1.17 - 2.03	1.97E-03
	PUMILIO2	2,800	542	18,628	1,996	0.55	0.50 - 0.61	4.30E-30		PUMILIO2	407	49	21,021	2,489	0.98	0.73 - 1.33	9.13E-01
16	AGO1	3,038	306	18,390	2,232	1.20	1.06 - 1.37	3.55E-03	22	AGO1	790	80	20,638	2,458	1.18	0.93 - 1.49	1.73E-01
	AGO2	1,227	105	20,201	2,433	1.41	1.15 - 1.73	9.53E-04		AGO2	260	25	21,168	2,513	1.23	0.82 - 1.87	3.16E-01
	AGO3	4,057	393	17,371	2,145	1.27	1.14 - 1.43	2.39E-05		AGO3	925	88	20,503	2,450	1.26	1.01 - 1.57	4.43E-02
	AGO4	3,314	242	18,114	2,296	1.74	1.51 - 1.99	1.90E-15		AGO4	540	43	20,888	2,495	1.50	1.10 - 2.05	1.07E-02
	PUMILIO2	1,579	314	19,849	2,224	0.56	0.50 - 0.64	9.90E-19		PUMILIO2	383	46	21,045	2,492	0.99	0.72 - 1.34	9.28E-01
17	AGO1	1,929	199	19,499	2,339	1.16	1.00 - 1.35	5.18E-02	23	AGO1	655	66	20,773	2,472	1.18	0.91 - 1.53	2.03E-01
	AGO2	678	55	20,750	2,483	1.48	1.12 - 1.95	5.81E-03		AGO2	232	23	21,196	2,515	1.20	0.78 - 1.84	4.13E-01
	AGO3	2,578	232	18,850	2,306	1.36	1.18 - 1.57	1.88E-05		AGO3	706	71	20,722	2,467	1.18	0.92 - 1.52	1.81E-01
	AGO4	1,934	147	19,494	2,391	1.61	1.36 - 1.92	4.49E-08		AGO4	443	34	20,985	2,504	1.55	1.09 - 2.21	1.31E-02
	PUMILIO2	960	166	20,468	2,372	0.67	0.57 - 0.79	3.51E-06		PUMILIO2	346	43	21,082	2,495	0.95	0.69 - 1.31	7.64E-01
18	AGO1	1,462	147	19,966	2,391	1.19	1.00 - 1.42	4.97E-02	24	AGO1	557	58	20,871	2,480	1.14	0.87 - 1.50	3.44E-01
	AGO2	483	35	20,945	2,503	1.65	1.17 - 2.33	4.15E-03		AGO2	210	21	21,218	2,517	1.19	0.76 - 1.86	4.57E-01
	AGO3	1,927	172	19,501	2,366	1.36	1.16 - 1.60	1.88E-04		AGO3	560	53	20,868	2,485	1.26	0.95 - 1.67	1.13E-01
	AGO4	1,352	108	20,076	2,430	1.52	1.24 - 1.85	4.29E-05		AGO4	367	29	21,061	2,509	1.51	1.03 - 2.21	3.31E-02
	PUMILIO2	686	96	20,742	2,442	0.84	0.68 - 1.05	1.19E-01		PUMILIO2	317	39	21,111	2,499	0.96	0.69 - 1.35	8.22E-01
19	AGO1	1,213	121	20,215	2,417	1.20	0.99 - 1.45	6.34E-02	25	AGO1	464	36	20,964	2,502	1.54	1.09 - 2.16	1.28E-02
	AGO2	398	30	21,030	2,508	1.58	1.09 - 2.30	1.51E-02		AGO2	189	20	21,239	2,518	1.12	0.71 - 1.78	6.30E-01
	AGO3	1,614	147	19,814	2,391	1.32	1.11 - 1.58	1.49E-03		AGO3	440	44	20,988	2,494	1.19	0.87 - 1.62	2.79E-01
	AGO4	1,049	83	20,379	2,455	1.52	1.21 - 1.92	2.63E-04		AGO4	305	26	21,123	2,512	1.40	0.93 - 2.09	1.03E-01
	PUMILIO2	523	70	20,905	2,468	0.88	0.68 - 1.14	3.30E-01		PUMILIO2	299	35	21,129	2,503	1.01	0.71 - 1.44	9.47E-01
20	AGO1	1,037	101	20,391	2,437	1.23	1.00 - 1.51	5.41E-02									
	AGO2	350	27	21,078	2,511	1.54	1.04 - 2.29	2.92E-02									
	AGO3	1,372	129	20,056	2,409	1.28	1.06 - 1.54	9.45E-03									
	AGO4	869	67	20,559	2,471	1.56	1.21 - 2.01	5.00E-04									
	PUMILIO2	439	61	20,989	2,477	0.85	0.65 - 1.11	2.37E-01									

Approximate match

Binding Length	Protein	a	b	c	d	OR	95 %CI	*p*-value	Binding Length	Protein	a	b	c	d	OR	95 %CI	*p*-value
15	AGO1	17,795	1,703	3,633	835	2.40	2.19 - 2.63	1.02E-84	21	AGO1	3,940	432	17,488	2,106	1.10	0.98 - 1.22	9.20E-02
	AGO2	12,918	863	8,510	1,675	2.95	2.70 - 3.21	1.62E-141		AGO2	894	99	20,534	2,439	1.07	0.87 - 1.33	5.17E-01
	AGO3	20,003	2,072	1,425	466	3.16	2.82 - 3.54	4.01E-95		AGO3	5,155	520	16,273	2,018	1.23	1.11 - 1.36	6.36E-05
	AGO4	19,180	1,715	2,248	823	4.09	3.73 - 4.50	1.48E-214		AGO4	1,804	158	19,624	2,380	1.38	1.17 - 1.64	1.38E-04
	PUMILIO2	14,456	1,580	6,972	958	1.26	1.15 - 1.37	1.34E-07		PUMILIO2	1,425	265	20,003	2,273	0.61	0.53 - 0.70	1.74E-12
16	AGO1	16,118	1,496	5,310	1,042	2.11	1.94 - 2.30	4.47E-69	22	AGO1	3,338	381	18,090	2,157	1.04	0.93 - 1.17	4.57E-01
	AGO2	10,357	685	11,071	1,853	2.53	2.31 - 2.77	1.73E-92		AGO2	717	85	20,711	2,453	1.00	0.79 - 1.26	9.94E-01
	AGO3	19,025	1,920	2,403	618	2.55	2.31 - 2.82	2.80E-79		AGO3	4,060	418	17,368	2,120	1.19	1.06 - 1.32	2.46E-03
	AGO4	17,729	1,458	3,699	1,080	3.55	3.26 - 3.87	9.94E-200		AGO4	1,252	121	20,176	2,417	1.24	1.02 - 1.50	2.75E-02
	PUMILIO2	12,752	1,430	8,676	1,108	1.14	1.05 - 1.24	2.14E-03		PUMILIO2	1,125	215	20,303	2,323	0.60	0.51 - 0.70	2.41E-11
17	AGO1	13,188	1,247	8,240	1,291	1.66	1.53 - 1.80	1.33E-33	23	AGO1	2,825	340	18,603	2,198	0.98	0.87 - 1.11	7.65E-01
	AGO2	6,763	462	14,665	2,076	2.07	1.87 - 2.30	1.01E-43		AGO2	589	76	20,839	2,462	0.92	0.72 - 1.17	4.76E-01
	AGO3	16,885	1,663	4,543	875	1.96	1.79 - 2.14	1.23E-51		AGO3	3,166	347	18,262	2,191	1.09	0.97 - 1.23	1.37E-01
	AGO4	14,358	1,074	7,070	1,464	2.77	2.55 - 3.01	3.28E-133		AGO4	967	96	20,461	2,442	1.20	0.97 - 1.49	9.11E-02
	PUMILIO2	9,999	1,205	11,429	1,333	0.97	0.89 - 1.05	4.36E-01		PUMILIO2	976	187	20,452	2,351	0.60	0.51 - 0.71	4.47E-10
18	AGO1	9,490	932	11,938	1,606	1.37	1.26 - 1.49	3.59E-13	24	AGO1	2,395	306	19,033	2,232	0.92	0.81 - 1.04	1.85E-01
	AGO2	3,650	279	17,778	2,259	1.66	1.46 - 1.89	7.68E-15		AGO2	504	65	20,924	2,473	0.92	0.71 - 1.19	5.13E-01
	AGO3	13,149	1,267	8,279	1,271	1.59	1.47 - 1.73	8.63E-29		AGO3	2,462	272	18,966	2,266	1.08	0.95 - 1.23	2.47E-01
	AGO4	9,281	658	12,147	1,880	2.18	1.99 - 2.40	2.03E-63		AGO4	764	86	20,664	2,452	1.05	0.84 - 1.32	6.49E-01
	PUMILIO2	6,505	920	14,923	1,618	0.77	0.70 - 0.84	1.29E-09		PUMILIO2	876	172	20,552	2,366	0.59	0.50 - 0.69	3.76E-10
19	AGO1	6,693	666	14,735	1,872	1.28	1.16 - 1.40	2.51E-07	25	AGO1	2,034	269	19,394	2,269	0.88	0.77 - 1.01	7.37E-02
	AGO2	1,964	173	19,464	2,365	1.38	1.17 - 1.62	8.61E-05		AGO2	436	57	20,992	2,481	0.90	0.68 - 1.20	4.79E-01
	AGO3	9,348	910	12,080	1,628	1.38	1.27 - 1.51	7.39E-14		AGO3	1,897	229	19,531	2,309	0.98	0.85 - 1.13	7.76E-01
	AGO4	5,046	370	16,382	2,168	1.80	1.61 - 2.02	1.66E-24		AGO4	625	70	20,803	2,468	1.06	0.82 - 1.36	6.52E-01
	PUMILIO2	3,739	590	17,689	1,948	0.70	0.63 - 0.77	7.04E-13		PUMILIO2	801	154	20,627	2,384	0.60	0.50 - 0.72	1.40E-08
20	AGO1	4,877	520	16,551	2,018	1.14	1.03 - 1.27	9.59E-03									
	AGO2	1,242	129	20,186	2,409	1.15	0.95 - 1.38	1.43E-01									
	AGO3	6,632	661	14,796	1,877	1.27	1.16 - 1.40	3.79E-07									
	AGO4	2,843	233	18,585	2,305	1.51	1.32 - 1.74	5.85E-09									
	PUMILIO2	2,111	367	19,317	2,171	0.65	0.57 - 0.73	5.58E-13									

2. Supplementary figures legends

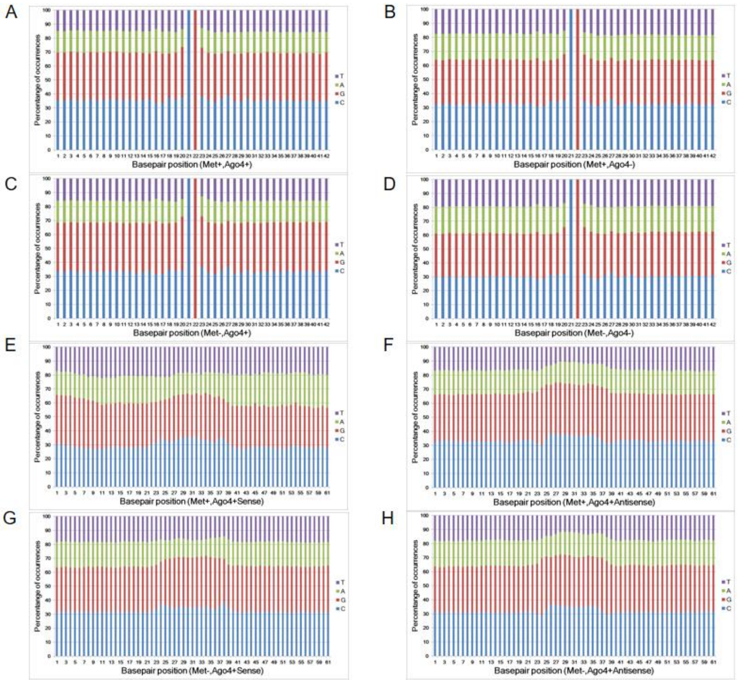

Figure S1 Distribution of DNA bases on methylation and AGO4 binding sites. Raw data of promoter methylation arrays provided both genomic location and methylation levels through 15 probes per one gene promoter. Each probe is 50 bp in length. Met+ is referred to as the methylation level in the 80th percentile. Remaining methylation levels were referred to as Met-. ClipZ database provided the genomic locations of AGO binding sites. Each binding site is about 15-20 bp in length. AGO4+ is referred to as the overlab between an AGO4 binding site and a methylation probe, while AGO4- is referred to as no AGO4 binding sites on a methylation probe. **(A to D)** The distribution categorized by (Met+, AGO4+), (Met+, AGO4-), (Met-, AGO4+), and (Met-, AGO4-), respectively. Sense strands were collected from every CpG on the methylation probes by centering CpG between 20-bp flanking sequences on the left and on the right. A total length was 42 bp. **(E to H)** The distribution categorized by (Met+, AGO4+sense), (Met+, AGO4+Antisense), (Met+, AGO4+Antisense), (Met-, AGO4+Sense), and (Met-, AGO4+Antisense), respectively where "Sense/Antisense" denotes the orientation of AGO4 binding sites. The sense strands of AGO4 binding sites were centered and then padded with flanking sequences. A total length was 61bp.

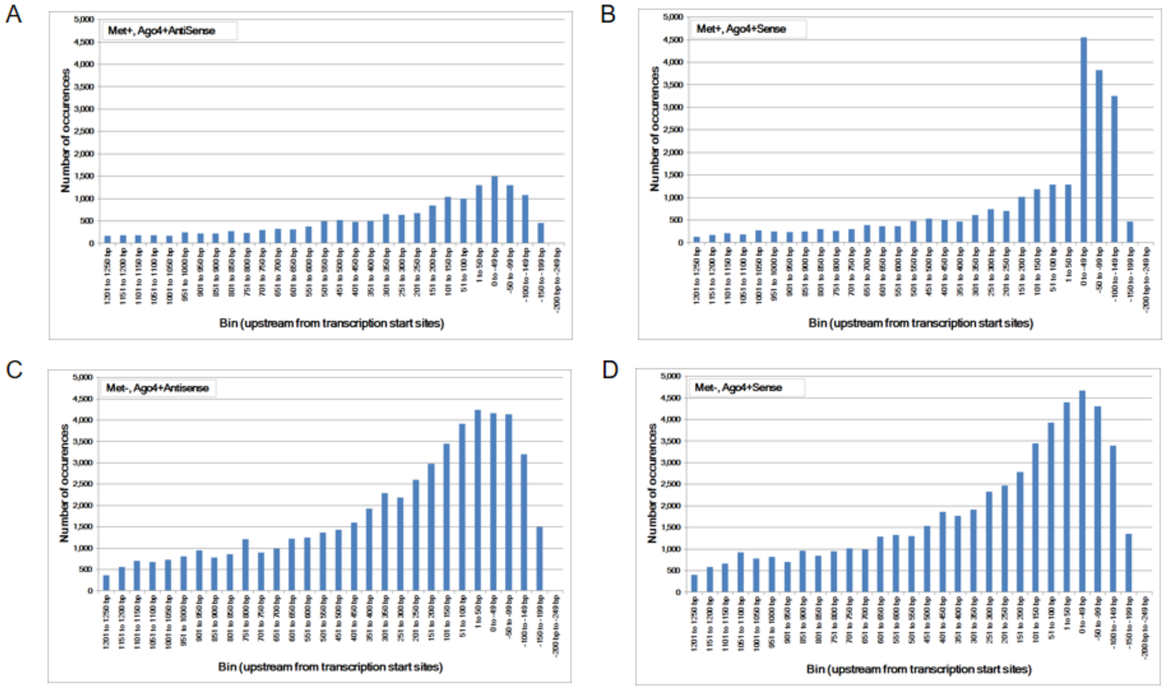

Figure S2 Distribution of AGO4 binding sites on gene promoters. **(A to D)** Distribution of (Met+,AGO4+antisense), (Met+,AGO4+sense), (Met-,AGO4+antiense), and (Met-,AGO4+sense), respectively. Definitions of Met+, Met-, AGO4+, AGO4-, sense, and antisense are given in supplementary figure S1.

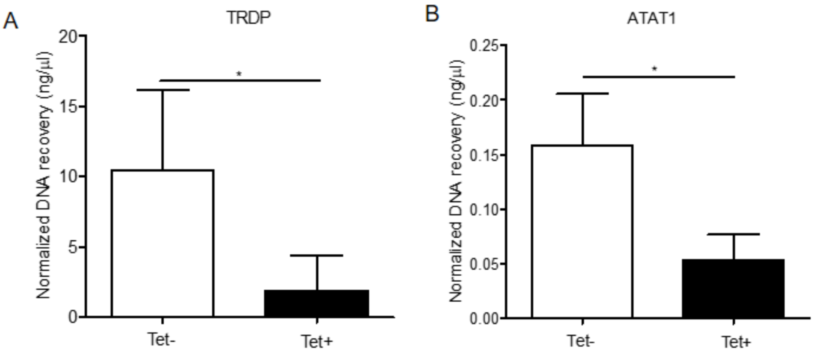

Figure S3 Proving of AGO4-binding genes. The graphs showed AGO4 quantities compared between the presence and absence of AGO4 in HEK293 cells, respectively. In Tet- condition, it significantly showed a higher amount of AGO4-binding genes than Tet+ condition **(A)** *TRDP* and **(B)** *ATAT1*, tested by a paired-sample T-test: $p<0.05$ is represented as *.

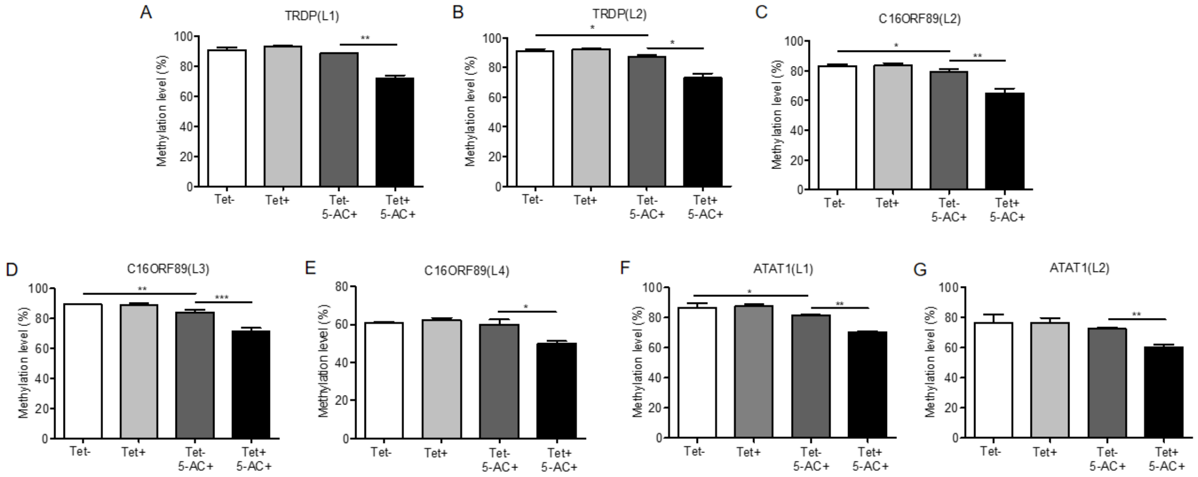

Figure S4 Confirmation of AGO4 protein associated to methylation level in AGO4-binding genes, independently function to DNA methyltransferase (DNMT). Each CpG location (represented as L) found in genes of interest was observed. Whether or not presense of AGO4 (Tet-, Tet+), methylation level in every gene did not change; however, when 5'-azacytidine was applied with the presence of AGO4 (Tet-, Aza+), methylation level in AGO4-binding genes was significantly promoted compared to the absence of AGO4 (Tet+, Aza+) **(A to B)** *TRDP*(L1) and (L2) **(C to E)** *C16ORF89*(L2), (L3) and (L4), respectively **(F to G)** *ATAT1*(L1) and (L2). Statistics was analyzed using a paired-sample T-test: $p<0.05$, $p<0.01$ and $p<0.005$ are represented *, ** and ***, respectively.

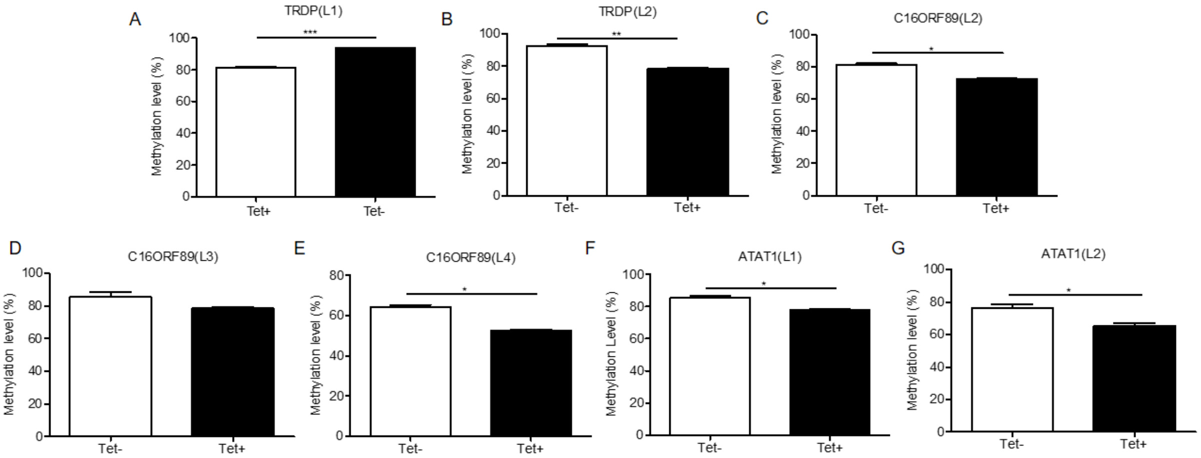

Figure S5 Recovery of methylation level in some gene-containing AGO4-binding sites, in concordance with the presence of the AGO4 proteins after withdrawal of 5'-azacytidine. The graphs depicted that methylation level was increased in Tet+ treated HEK293 cells, statistical test using a paired-sample T-test was shown where $p<0.05$, $p<0.01$ and $p<0.005$ are represented as *, ** and ***, respectively. **(A to B)** *TRDP*(L1) and (L2) **(C to E)** *C16ORF89*(L2), (L3) and (L4) **(F to G)** *C6ORF134*(L1) and (L2).

Tet 5-AC and combined Tet+5-AC

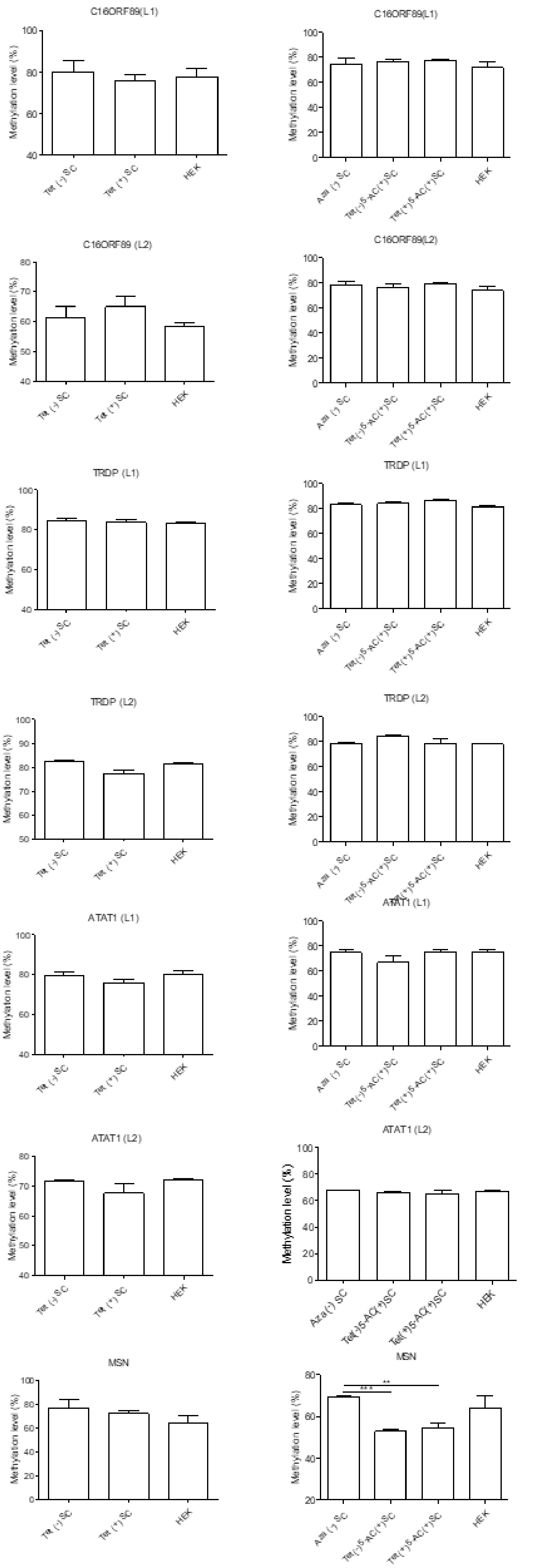

Figure S6 Tetracycline (Tet) (the first panel), and 5'-azacytidine and combined tetracycline and 5'-azacytidine treatment (the second panel) in scramble shRNA transfected HEK293 cells. Methylation levels were observed in AGO4-binding genes: *C16ORF89* (L1 to L2), *TRDP* (L1 and L2), *ATAT1* (L1 and L2) and non AGO4-binding gene, *MSN*. Statistical test using an unpaired T-test was shown where $p<0.05$ is represented as **, and SC means scramble shRNA transfected cells.

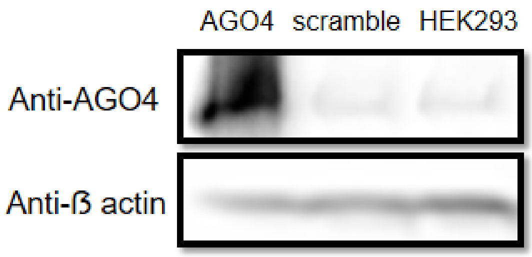

Figure S7 AGO4 protein expression in AGO4 plasmid and scramble shRNA transfected- and untransfected HEK293 cells at 48 hours after transfection. AGO4 protein was upregulated in AGO4 plasmid transfected HEK293 while scramble shRNA transfected HEK293 showed low level of AGO4 protein as same as in untransfected HEK293 cells. β-actin was used to confirm equal protein loading of each lane.
